# Brain stem tumors in children less than 3 months: Clinical and radiologic findings of a rare disease

**DOI:** 10.1007/s00381-023-06272-w

**Published:** 2024-02-20

**Authors:** Danai Papangelopoulou, Brigitte Bison, Lars Behrens, Simon Bailey, Marc Ansari, Karoline Ehlert, Ofelia Cruz Martinez, Christof M. Kramm, Andres Morales La Madrid, Andre O. von Bueren

**Affiliations:** 1 Department of Pediatrics, Gynecology and Obstetrics, Division of General Pediatrics, Pediatric Hematology and Oncology Unit, University Hospitals of Geneva, Geneva, Switzerland; 2 Cansearch Research Platform for Pediatric Oncology and Hematology, Faculty of Medicine, Department of Pediatrics, Gynecology and Obstetrics, University of Geneva, Geneva, Switzerland; 3 Diagnostic and Interventional Neuroradiology, Faculty of Medicine, University of Augsburg, Augsburg, Germany; 4 Wolfson Childhood Cancer Research Centre, Newcastle University Centre for Cancer, Newcastle upon Tyne, UK; 5 Department of Pediatric Hematology and Oncology, University Medicine Greifswald, Greifswald, Germany; 6 Pediatric Cancer Center Barcelona, Hospital Sant Joan de Déu, Barcelona, Spain; 7 Department of Pediatrics and Adolescent Medicine, Division of Pediatric Hematology and Oncology, University Medical Center Goettingen, Goettingen, Germany

**Keywords:** Brain stem tumors, Diffuse midline glioma (DMG), Neonatal, Congenital

## Abstract

**Purpose:**

Brain stem tumors in children < 3 months at diagnosis are extremely rare. Our aim is to study a retrospective cohort to improve the understanding of the disease course and guide patient management.

**Methods:**

This is a multicenter retrospective analysis across the European Society for Pediatric Oncology SIOP-E HGG/DIPG Working Group linked centers, including patients with a brainstem tumor diagnosed between 2009 and 2020 and aged < 3 months at diagnosis. Clinical data were collected, and imaging characteristics were analyzed blindly and independently by two neuroradiologists.

**Results:**

Five cases were identified. No patient received any therapy. The epicenter of two tumors was in the medulla oblongata alone and in the medulla oblongata and the pons in three. For patients with tumor in equal parts in the medulla oblongata and the pons (*n* = 3), the extension at diagnosis involved the spinal cord; for the two patients with the tumor epicenter in the medulla oblongata alone (*n* = 2), the extension at diagnosis included the pons (*n* = 2) and the spinal cord (*n* = 1). Biopsy was performed in one patient identifying a pilocytic astrocytoma. Two patients died. In one patient, autopsy revealed a high-grade glioma (case 3). Three survivors showed either spontaneous tumor regression (*n* = 2) or stable disease (*n* = 1). Survivors were followed up for 10, 7, and 0.6 years, respectively. One case had the typical imaging characteristics of a dorsal exophytic low-grade glioma.

**Conclusions:**

No patient fulfilled the radiologic criteria defining a high-grade glioma. Central neuroradiological review and biopsy may provide useful information regarding the patient management.

**Supplementary Information:**

The online version contains supplementary material available at 10.1007/s00381-023-06272-w.

## Introduction

Pediatric brainstem tumors including brain stem gliomas account for 10–20% of all central nervous system pediatric neoplasms [1]. The mean age at diagnosis is 7–9 years [2] but may occur at any age. Approximately 80% of pediatric brainstem gliomas arise within the pons, while the remaining 20% arise in the medulla, midbrain, or cervicomedullary junction [3]. Approximately 80% of those arising in the pons are diffuse intrinsic pontine gliomas (DIPGs). In these cases, with typical imaging characteristics and the classic clinical triad of long tract signs with motor weakness, cranial neuropathies, and cerebellar signs, therapy is often initiated without histological and/or molecular confirmation. In all other cases of brainstem tumors, a biopsy is mandatory for diagnosis [4]. Biopsy has now emerged as a routine standard of care in many institutions in DIPG at presentation [5–7]. Up to 90% of DIPGs harbor a point mutation in H3F3A (65% of tumors) or HIST1H3B (25% of tumors), the remaining 10% of patients have a histone 3 wild-type tumor [6, 8–10].

Brain tumors in infants differ in clinical presentation, anatomical distribution, histopathological diagnosis, therapy, and prognosis from those occurring in older children. The diagnosis of brain tumors in patients less than 12 months is usually delayed by the lack of symptoms and verbal complaints [11]. Macrocephaly, changes of behavior, and delayed developmental milestones are usual clinical findings [12]. The most common tumors in children aged less than 3 months are high-grade glioma (19.3%), teratoma (17.5%), and ungraded glioma (14.6%), while in the 3 to 6 months age group, low-grade glioma (18.9%), high-grade glioma (14.4%), and central nervous system (CNS) atypical teratoid/rhabdoid tumor (AT/RT) (13.9%) [13].

Brain stem tumors occurring in children aged less than 3 months at diagnosis are extremely rare. Primary brain tumors detected in utero or during early postnatal life are estimated to affect 1.1–3.4 per million live births [14, 15]. Given the rarity and absence of clinical trial data in children aged less than 6–12 months, the knowledge about diagnosis and management of tumors in this age group is limited. We report five cases of infants aged < 3 months at diagnosis with a brainstem tumor who were diagnosed between 2009 and 2020. We wish to improve the understanding of presentation and patient management regarding diagnosis and treatment of brain stem tumors in this age group.

## Methods and patients

### Study design and inclusion criteria

This is a multicenter retrospective analysis across the European Society for Pediatric Oncology (SIOP-E) HGG/DIPG Working Group linked centers. Inclusion criteria were defined as children aged less than three months at diagnosis with brainstem tumor, diagnosed between 2009 and 2020, and with informed consent signed by the legal guardians of the patients. Between 2009 and 2020, five eligible children aged less than 3 months at diagnosis with a brainstem tumor fulfilled the inclusion criteria and were included in this study.

### Data collections and analysis

Coded health-related personal and clinical data of patients aged less than 3 months with brainstem tumors treated at SIOP-E (Société Internationale d’Oncologie Pédiatrique) linked centers were collected by each co-investigator. All patients/legal representatives signed consent to have their child’s data collected and used for this study. Five children were included in this report (Spain, *n* = 2; Germany, *n* = 2; UK, *n* = 1). Each co-investigator filled out an excel spreadsheet with 17 variables (the complete list of variables is available in Supplementary Table 1), after investigating the medical file of every patient. Original clinical, laboratory, and imaging data were included. Data were recorded on gender, medical history, presenting symptoms, cerebrospinal fluid analysis, biopsy/autopsy findings, treatment details, clinical evolution, survival, and follow-up time. These coded data were then verified by two authors. Coded imaging data (magnetic resonance imaging) were further analyzed by two experienced neuroradiologists (blind to clinical information) in an independent manner. The following radiologic variables were recorded: epicenter, extension at diagnosis, contact with the surface of the brain, brainstem enlargement, brain stem segment involved, tumor margin, enhancement characteristics, the presence of necrosis/hemorrhage/edema, the presence of metastases, and the presence/absence of hydrocephalus.

### Statistical analyses

Overall survival (OS) (days) was defined as the time from date of diagnosis to death (or date of last follow-up). Descriptive data were presented as frequencies and percentages.

### Ethical considerations

Ethical approval was obtained from the institutional board of the Geneva Research Ethics Committee (*Commission cantonale d’éthique de la recherche*). The assigned protocol number is 2022-01599.

## Results

### Baseline characteristics

Patients’ key characteristics are displayed in Table 1 (complete patients’ characteristics are illustrated in Supplementary Table 1). The median age at diagnosis was 18 days (range 0–42 days) of life; with prenatal imaging providing the diagnosis in one case. The symptoms were evident in all but one child at birth. Case 3 developed symptoms during the first month of life. All patients presented with hypotonia and frequently (*n* = 3) with respiratory distress. The median interval between onset of symptoms and diagnosis was 18 days.

**Table 1:** Patients’ characteristics

**Patients**	**1**	**2**	**3**	**4**	**5**
**Gender**	Female	Female	Male	Female	Female
**Age at diagnosis**	18 days	1st day	42 days	Prenatal	19 days
**Symptoms**	Poor feeding, hypotonia	Respiratory distress, hypotonia	Respiratory distress, hypotonia	Respiratory distress, hypotonia	Poor feeding, nystagmus, hypotonia
**Date of onset symptoms**	At birth	At birth	1st month of life	At birth	At birth

### MRI characteristics

**Fig. 1:**
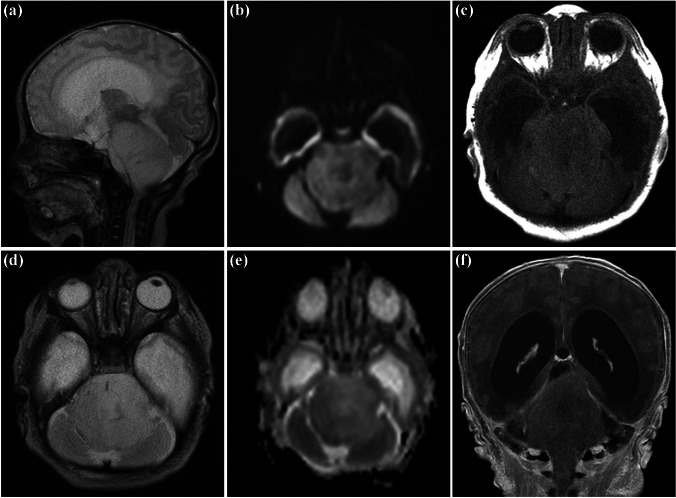
Case 2. First MRI at the second day of life. Sagittal (**a**) and axial (**d**) T2-weighted images showing a confluent mass expanding pons and medulla oblongata and occluding the foramen magnum. Mildly increased diffusion on diffusion-weighted imaging (iso- to hypointense signal on b1000 (**b**) and bright signal on apparent diffusion coefficient map (**e**), no contrast enhancement, axial T1-weighted image before (**c**), and coronal T1-weighted image after application of gadolinium-based contrast agent (**f**). Dilated supratentorial ventricles and no signs of dissemination

**Fig. 2:**
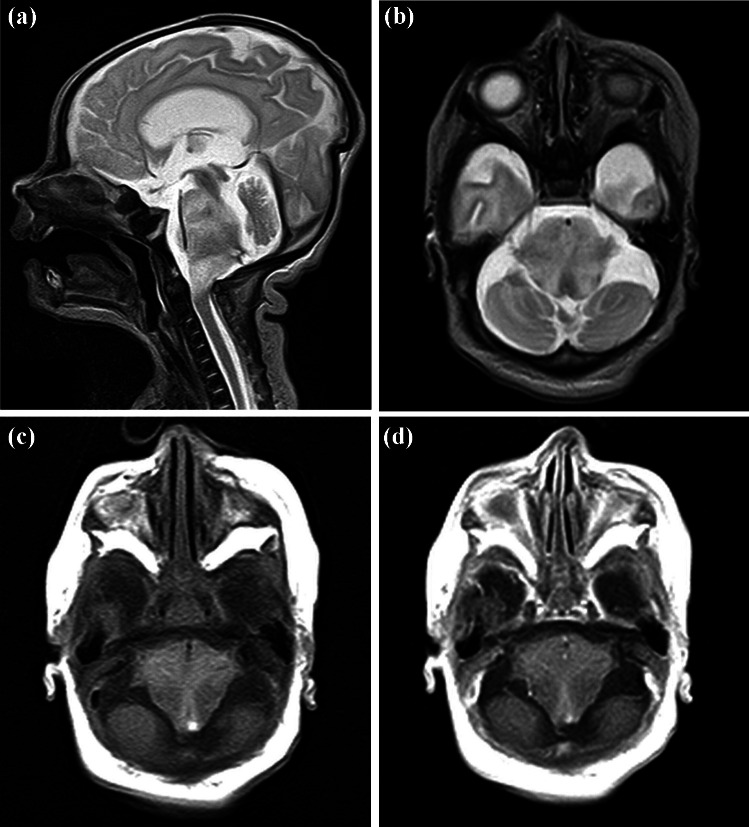
Case 4. First postnatal MRI at the twelfth day of life with sagittal (**a**) and axial (**b**) T2-weighted images demonstrating the diffuse infiltrating tumor with moderate expansion in equal parts in pons and medulla oblongata, no enhancement after application of gadolinium-based contrast agent (T1-weighted images without (**c**) and with contrast agent (**d**), both axial planes)

**Fig. 3:**
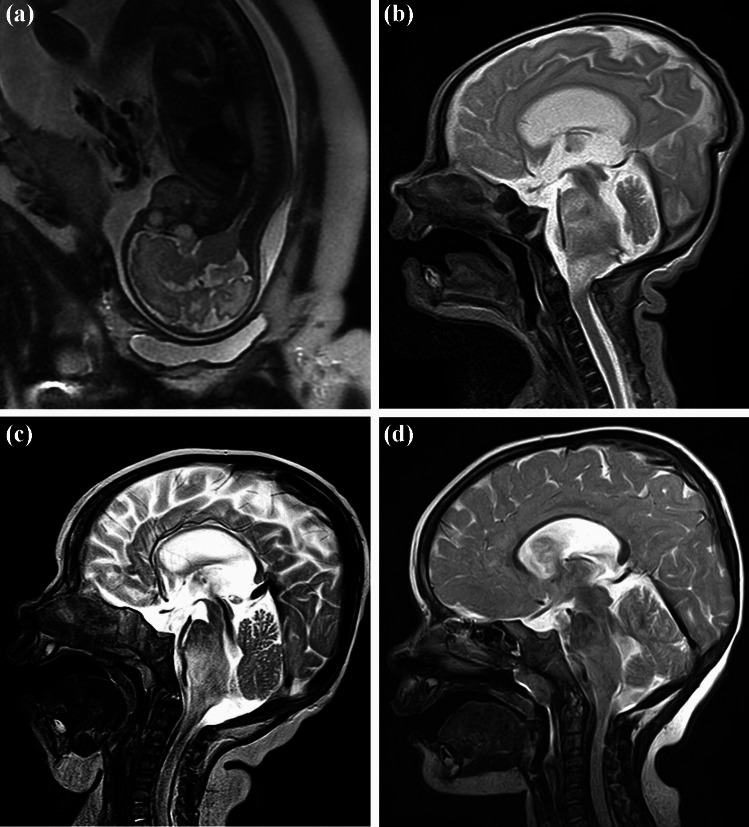
Case 4. Follow-up. All T2-weighted images, sagittal plane; intrauterine MRI obtained 24 days before birth (**a**), at the 12th day of life (**b**), at the age of 5 months (**c**), and 2 years old (**d**). After detecting the tumor with ultrasound, the brainstem glioma is already visible prenatally on MRI, demonstrating an increased volume at day 12 of life and a spontaneous and continues regression documented until the second year of life

**Fig. 4:**
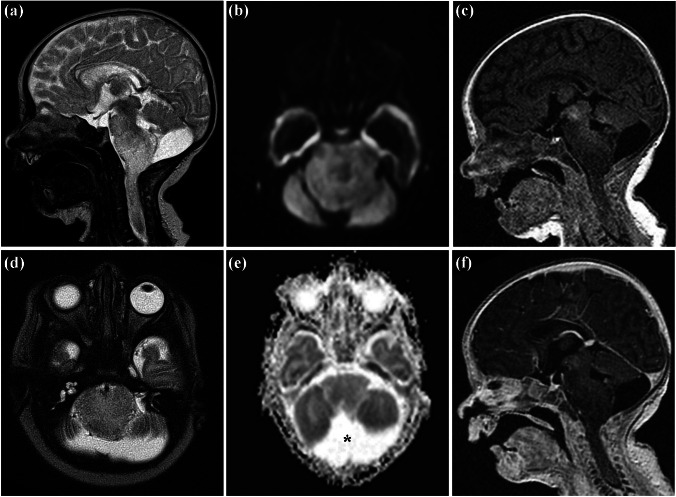
Case 5. First MRI at the age of 19 days. Sagittal (**a**) and axial (**d**) T2-weighted images showing a diffuse infiltrating tumor centered in the medulla oblongata, having a dorsal exophytic component, extending into the pons and the upper spinal cord. The foramen magnum is occluded. No change of diffusion on diffusion-weighted image (**b**) or apparent diffusion coefficient-map (**e**), no contrast enhancement visible on T1-weighted images before (**c**) and after application of a gadolinium-based contrast agent (**f**), both sagittal planes. Regular width of the ventricles, no dissemination detected. Mega cisterna magna as an incidental finding (asterisk)

Initial cranial MRI studies were available for all five patients and centrally reviewed by two experienced neuroradiologists (Figs. 1, 2, 3 and 4), summarized in Table 2. At diagnosis, a cerebral MRI was performed for every patient with additional spinal MRI available for cases 1 and 4. The epicenter of two tumors was in the medulla oblongata alone and in the medulla oblongata and the pons in three tumors. For all patients with equal parts of the tumor in the medulla oblongata and the pons (*n* = 3), the extension at diagnosis included the spinal cord; for the two patients with epicenter in the medulla oblongata alone (*n* = 2), the extension at diagnosis involved the pons (*n* = 2) and the spinal cord (*n* = 1). Brainstem enlargement was marked in all patients. Tumor margins were able to be delineated in three patients. There was no evidence of metastatic spread in any patient. Only one patient’s tumor was dorsal exophytic (case 5). Hydrocephalus was documented in four patients (severe hydrocephalus; case 2). No peritumoral edema nor necrosis was seen in any of the initial MRI studies. There were no obvious residues reacted to bleeding, although this was unclear for one patient (case 4). All five tumors showed a predominantly low signal on T1-weighted images, three of them with a heterogeneous signal. Most of the tumors (*n* = 4) showed a bright signal on T2-weighted images, although it was heterogeneous in four cases. Macroscopic leptomeningeal dissemination could be excluded in three patients, only cranial dissemination could be excluded in cases 2 and 3, as no spinal MRI was performed. No scan demonstrated the definite imaging diagnosis of a pontine diffuse midline glioma (DIPG). Case 3 had an isointense signal on T2-weighted images, suggestive of high cellularity, although this could not be confirmed radiologically, as diffusion-weighted images were not provided. All cases were radiologically compatible with low-grade glioma. The tumor type in case 5 was confirmed by histology. Two patients died with a rapidly expanding tumor.

**Table 2:** Review of brain MRI studies obtained at diagnosis

**Case no.**	**MRI**	**Epicenter**	**Extension at diagnosis**	**Brainstem enlargement**	**Involved brainstem segment**	**Tumor margin**	**Signal T1**	**Signal T2**	**Contrast enhancement (%)**	
**1**	Brain and spinal	Med obl	Pons	Marked	Med obl > pons	Blurred	Hypo/hetero	Hyper/hetero		Multifocal (≤ 25%)
**2**	Brain	Med obl and pons	Spinal cord	Marked	Med obl = pons	Mostly delineated	Hypo/hetero	Ηyper/hetero		Focal (≤ 10%)
**3**	Brain	Med obl and pons	Spinal cord	Marked	Med obl = pons	Mostly delineated	Hypo/homo	Iso/homo		None
**4**	Brain and spinal	Med obl and pons	Spinal cord	Marked	Med obl = pons	Mostly delineated	Hypo/hetero	Hyper/hetero		Focal (≤ 10%)
**5**^**a**^	Brain	Med obl	Pons & Spinal cord	Marked	Med obl > pons	Blurred	Hypo/homo	Hyper/hetero		Focal (≤ 10%)

*MRI* magnetic resonance imaging, *Med obl* medulla oblongata, *Hypo* hypointense, *Homo* homogeneous, *Hetero* heterogeneous

^**a**^Dorsal exophytic

### Survival and further clinical course of the patients

All five patients underwent observation and best supportive care (Table 3). No patient underwent tumor resection, and no patient received any tumor directed therapy. Two patients died (overall survival; 9 days, case 2; 66 days, case 3). Biopsy was performed in one patient; this analysis revealed a pilocytic astrocytoma (case 5) with strongly positive staining for GFAP. There was an absence of IDH1 p.R132H expression, and H3 p.K28M (K27M) immunohistochemistry was negative. The Ki-67 index was approximatively 3%. Autopsy was performed in one patient (case 3) showing a high-grade glioma not otherwise specified (NOS), with necrotic elements that made immunohistochemistry results difficult to interpret. We noticed spontaneous regression (*n* = 1, case 1), partial regression of the tumor (*n* = 1, case 4), and stability of the disease (*n* = 1, case 5). The surviving patients were followed up for 10, 7, and 0.6 years, respectively.

**Table 3:** Patients’ further clinical course

**Patients**	**1**	**2**	**3**	**4**	**5**
**Biopsy**	No	No	No	No	Pilocytic astrocytoma (grade I)
**Autopsy**	No	No	High-grade glioma^a^	No	No
**Treatment**	Observation	Observation	Observation	Observation	Observation
**Progression of disease**	Complete remission	Progressive disease	Progressive disease	Partial remission	Stable disease
**Vital status**	Alive	Dead	Dead	Alive	Alive
**Follow-up**	10 years	9 days	2 months	7 years	7 months

^a^Glial infiltration of pons and medulla. Necrotic elements made immunohistochemistry results inconclusive

## Discussion

Pediatric brain stem gliomas are a heterogeneous group of entities and may be classified based on their clinical and MRI appearances [16, 17]. Brain stem tumors in children less than three months remain extremely rare and result in a challenging situation. Little is known in the literature about brain stem tumors appearing in this age group. Our aim was to study a retrospective cohort of this limited age group to improve the understanding of these cases and subsequent patient management.

In our multicenter retrospective study, we identified five cases of brain stem tumors in children aged < 3 months. After MRI review by two experienced neuroradiologists, no case fulfilled the diagnostic imaging criteria of a DIPG. One demonstrated the typical appearance of a dorsal exophytic brainstem low-grade glioma and the rest compatible with low-grade gliomas of the brain stem. One case had an intermediate signal on T2-weighted images, suggesting higher cellularity (no diffusion-weighted images available). To aid diagnosis, a comprehensive MRI according to guidelines is required with advanced MRI techniques such as MR-spectroscopy and ASL-perfusion desirable [18]. The radiologic diagnosis was confirmed by histopathology in case 5, for case 1 and 4 the clinical course was in keeping with the radiologic diagnosis. However, this is less clear for cases 2 and 3. The patient in case 2 had a swollen pontomedullary tumor occluding the foramen magnum and compressed the brain at the level of the craniospinal junction. This patient had a rapid and fatal clinical course corresponding to an unusual evolution for a low-grade glioma patient. The histologic evaluation of the autopsy of case 3 suggested evidence for a high-grade glioma, although analysis was limited due to relatively low tissue and imaging quality.

There are few previous reports regarding imaging characteristics and clinical evolution in brainstem tumors during the first months of life. Pontine gliomas in neonates and young infants may include a variety of different entities with different biological behavior [19–22]. There have been a small number of case reports of neonates with brain stem tumors (Table 4). However, the patients included had very different outcomes [23–30].

**Table 4:** Published cases of brain stem tumors in children less than 3 months identified in the literature

**Cases**	Age at Dx*	**Symptoms**	**Imaging features**	**Biopsy**	**Autopsy**	**Treatment**	**Vital status**	**Follow-up**
**Thomson and Kosnic et al.** **Case 1**	2 days	Inspiratory stridor, hypotonia, facial palsy, vertical nystagmus, and disconjugate gaze	Brain MRI on day 2: large expansive mass involving the brainstem, predominantly the pons and extending into the medulla. Hypointense on T1-weighted and hyperintense on T2-weighted images	No	No	Supportive care	Alive	4 years: brainstem improved but still abnormal contour
**Thomson and Kosnic et al.** **Case 2**	1 week	Facial palsy, hemiparesis, weak cough/gag, disconjugate gaze, and hypertonicity	Brain MRI at 1st week: large mass centered within the pons extending from the inferior medulla oblongata up to the midbrain. Infiltrative and heterogenous in signal	No	No	Supportive care	Alive	10 years: remarkable regression of the lesion with many areas of cystic degeneration Slight facial asymmetry, subtle dysmetria on the right side during finger-to-nose testing
**Schomerus et al.**	7 weeks	Severe postnatal asphyxia, hypotonia, and horizontal nystagmus	Cranial US scan at 7 weeks: enlarged and very echogenic pons Brain MRI: hydrocephalus with enlarged third and lateral ventricles and swollen pons Large areas of the pons hypointense on T1-weighted and hyperintense on T2 Small area of old hemorrhage within the left basal pons and signs of old periventricular hemorrhage in the left occipitoparietal region	No	No	VPS at 2 months	Alive	MRI at 27 months: pons no longer swollen, signs of bilateral periventricular leukomalacia. 38 months: motor development of a 3-month-old
**Airewele et al.**	11 weeks	6-week history of vomiting, progressive respiratory stridor, horizontal nystagmus, right facial nerve palsy, tongue fasciculation	Brain MRI: poorly enhancing mass arising from medulla and extending posteriorly to compress right side of 4th ventricle, mild hydrocephalus	No	No	No treatment	Alive	12 years: mass remained stable on MRI. Normal neurological examination
**Shah et al.** **Case 1**	Prenatal	Mild respiratory distress	Brain MRI on day 2: hydrocephalus and large intrinsic pontine mass extending to the midbrain, upper cervical cord, and posterior fossa. Low T1 and high T2 signals and lacked contrast enhancement. Spinal MRI: normal features	No	Yes: PNET	Dexamethasone	Dead	12 days
**Shah et al.** **Case 2**	2 days	Hypotonia, right facial palsy, stridor, abnormal eye movements, pooling of secretions and abnormal gag reflex	Brain CT: mass originating in the posterior fossa. Brain MRI on day 2: intrinsic pontine mass 3.5 cm in diameter with involvement of the medulla oblongata and the middle cerebellar peduncle	No	No	Dexamethasone	Dead	11 days
**Swenson et al.**	Prenatal	Severe respiratory distress, with clinical signs of increased intracranial pressure	Fetal MRI: expansile, poorly marginated brainstem lesion with abnormal T1 hypointensity and T2 hyperintensity. Centered in the pons and extended cephalad into the midbrain, caudally into the medulla and posteriorly into the cerebellar peduncles. Severe hydrocephalus	No	Yes: anaplastic oligodendroglioma, with focal areas of grade IV astrocytoma or ganglioglioma (low grade)	EVD and intravenous dexamethasone	Dead	3 days
**Gabel et al.**	4 days	Axial hypotonia, right esotropia, facial weakness, poor feeding and abnormal respirations	Brain MRI: large tumor centered in the pons without reduced diffusivity on diffusion-weighed sequences and absent gadolinium enhancement	No	No	Palliative care	Dead	7 days
**Suo-Palosaari et al.**	Prenatal	No abnormal neurological signs or symptoms	Fetal MRI: expansive brainstem tumor, dislocating the cerebellum posteriorly. Homogeneous and hyperintense on T2-weighted images. Lateral ventricles were marginally dilated	No	No	Supportive care	Alive	4 years: diameter of the tumor has not diminished, but the size of the tumor has clearly decreased. No neurological signs or symptoms
**Satrom et al.**	5 days	Inspiratory stridor, respiratory distress with sternal retractions, and a grade II/VI systolic murmur, normal neurologic examination	Brain MRI: abnormal, mass-like expansion with T2 signal abnormality of the pons and medulla	No	Yes: WHO grade III astrocytoma	Palliative care	Dead	First days of life

*Dx* diagnosis, *MRI* magnetic resonance imaging, *CT* computed tomography scanning, *US* ultrasound, *PNET* brainstem primitive neuroectodermal tumor, *VPS* ventriculoperitoneal shunt, *EVD* external ventricular drain

In the patients presented in this study, we observed spontaneous tumor regression without receiving any treatment in most cases (*n* = 2 and stable disease *n* = 1). This observation is well described in the literature as case reports [23, 26, 28, 30]; Thomson and Kosnik reported in 2005 two full-term newborns with cranial nerve palsies and limb weakness and with MRI features compatible with diffuse brainstem lesions. Surprisingly in both patients a spontaneous partial tumor regression without any treatment was observed [30]. Schomerus et al. reported in 2006 a full-term male neonate who presented with severe post-natal asphyxia, low muscle tone and horizontal nystagmus at birth. The MRI at 7 weeks of age showed hydrocephalus with enlarged third and lateral ventricles and a swollen pons. Spontaneous partial tumor regression was observed. At the age of 10 years, his clinical condition appears to be normal [26]. Airewele et al. in 2007 reported an 11-week-old female who presented with vomiting and respiratory stridor and whose MRI features demonstrated a relatively non-enhancing mass arising from the medulla oblongata and extending posteriorly to compress the right lateral aspect of the fourth ventricle. The girl received no treatment, and a follow-up MRI at 5 years showed reduction of the mass; this radiologic situation remained stable at a follow-up MRI 7 years later [23]. Suo-Palosaari et al. in 2016 presented a female newborn with a diffuse brainstem lesion diagnosed by fetal MRI and without biopsy confirmation. Follow-up MRI demonstrated regression of the mass extension without any treatment, the patient was well with no neurological deficit on last follow-up at the age of 4 years [28]. In our retrospective study, biopsy was performed in one patient (*n* = 1) with the histological diagnosis of pilocytic astrocytoma, in contrast with previously published cases where no biopsy was performed in any patient [23–30]. Most of our patients (*n* = 3) are alive at the time of this retrospective review. The autopsy of case 3 revealed a high-grade glioma (Table 3). Of the cases described in Table 4, autopsy was performed in three patients out of the five who died (*n* = 3) and these revealed the diagnosis of brainstem primitive neuroectodermal tumor (PNET), anaplastic oligodendroglioma with focal areas of grade IV astrocytoma or ganglioglioma (low-grade), and WHO grade III astrocytoma [25, 27, 29].

Our study has several limitations. We have incomplete staging as spinal MRI was performed in only two patients (cases 1 and 4). The reason for incomplete staging might be explicable by the aggressive clinical behavior with rapid lethal evolution in two patients (cases 2 and 3); thus, spinal MRI would not have changed the management of these two patients. A biopsy was performed only in case 5 revealing a pilocytic astrocytoma, and an autopsy in one of two patients died, without any additional molecular analysis available. More recently, biopsy has emerged as a routine consideration in DIPG at presentation, not only in the context of an approved clinical study, but also as part of routine care at many institutions. However, in this context, it appears not to be surprising that only one patient was biopsied. In our study, we could not use the fifth edition of the WHO Classification of Tumors of the Central Nervous System (WHO CNS5) published in 2021, incorporating numerous molecular changes with clinicopathologic utility [31], due to several reasons. The retrospective study was conducted between 2009 and 2020; the tumors were not all biopsied, and comprehensive molecular analyses were not assessed.

Surgery to obtain tissue for histological and molecular analysis in this population often leads to a difficult decision; surgery presents an increased risk of morbidity and mortality, although postmortem tumor donations are a valuable opportunity to collect tissue to investigate underlying tumor biology. The main reason for parents to refuse autopsy is their wish to avoid actions the child disliked while alive even after their death. Parental psychological distress represents the second most common cause for refusal, and religious reasons appeared to play only a secondary role in the parents’ decision making [32]. A qualitative study conducted in Australia by Robertson et al. showed that parents felt comforted knowing that the donation would help better understand their child’s tumor [33].

Our retrospective study is small, and we are unable to draw any definitive conclusions. For future patients, we recommend transferring these rare cases to specialized centers. Biopsy might be considered depending on the clinical situation. By investigating the tissue of such rare tumors, we may learn more about molecular features, treatment possibilities, and prognosis. Complete staging at diagnosis, central radiologic review of the imaging, and biopsy to perform pathologic and molecular analyses would help to better characterize and diagnose the disease and thus might be helpful for the patient management [34].

## Conclusions

In our retrospective analysis, no child fulfilled the radiologic criteria for diffuse intrinsic pontine gliomas. Spontaneous regression may be the case, so close clinical and radiologic follow-up is highly recommended especially in the absence of histologic/molecular confirmation of the aggressiveness of the lesion. Biopsy of brain stem tumors with complete molecular assessment combined with detailed radiologic evaluation might be helpful in the decision concerning patient management. A prudent approach in this age group with this symptom and radiologic findings should be considered carefully. A multidisciplinary approach for these challenging cases, including palliative care teams with end-of-life discussions and adequacy of interventions is critical in such fragile cases.

### Supplementary Information

Below is the link to the electronic supplementary material.

Supplementary file1 (DOCX 20 KB)
