# Supplementary material for: Brain stem tumors in children less than 3 months: Clinical and radiologic findings of a rare disease
Source: Childs Nerv Syst. 2024 Feb 20;40(4):1053–64. doi: 10.1007/s00381-023-06272-w (PMC10972984; doi:10.1007/s00381-023-06272-w)
Supplement: Supplementary file 1 — Supplementary file1 (DOCX 20 KB) [file 381_2023_6272_MOESM1_ESM.docx]

**Supplementary Table 1. Complete Patients’ characteristics**

| Patients | 1 | 2 | 3 | 4 | 5 |
| --- | --- | --- | --- | --- | --- |
| Gender | female | female | male | female | female |
| Year of birth | 2009 | 2011 | 2018 | 2013 | 2020 |
| Age at diagnosis | 18 days | prenatal | 42 days | prenatal | 19 days |
| Date of onset symptoms | at birth | at birth | 1st month of life | at birth | at birth |
| Symptoms | poor feeding, hypotonia | respiratory distress, hypotonia | respiratory distress, hypotonia | respiratory distress, hypotonia | poor feeding, nystagmus, hypotonia |
| Prenatal diagnosis | no | yes*^1^ | no | yes*^2^ | no |
| CSF analysis at diagnosis | no | no | no | yes  (no data) | yes  (no data) |
| First MRI | 18^th^ day | 1^st^ day | 42^nd^ day | 12^th^ day | 19^th^ day |
| Spinal MRI at diagnosis | yes | no | no | yes | no |
| Biopsy | no | no | no | no | pilocytic astrocytoma (Grade I) |
| Treatment | observation | observation | observation | observation | observation |
| Progression of disease | complete remission | progressive disease | progressive  disease | partial  remission | stable  disease |
| Age at progression | no data | no data | 66 days | no data | no data |
| Vital status | alive | dead | dead | alive | alive |
| Autopsy | no | no | high-grade glioma*^3^ | no | no |
| Molecular characterization | no | no | H3 WT; BRAF WT; WT HIST1 | no | no |
| Follow-up | 10 years | 9 days | 2 months | 7 years | 7 months |

CSF = Cerebrospinal fluid; MRI = Magnetic Resonance Imaging

*^1^ Prenatal ultrasonography shows polyhydramnios and enlarged lateral ventricles.

*^2^ Prenatal MRI shows wide lateral ventricles and mass effect of the pons.

*^3^ Glial infiltration of pons and medulla. Necrotic elements made immunohistochemistry results inconclusive.
